# Role of aggregate-forming pilus (AFP) in adherence and colonization of both intestinal and urinary tracts

**DOI:** 10.1080/21505594.2022.2112818

**Published:** 2022-08-18

**Authors:** Paulo A. Schüroff, Cecilia M. Abe, Jonatas W. Silva, Cidéli de Paula Coelho, Fernanda B. Andrade, Rodrigo T. Hernandes, Ulrich Dobrindt, Tânia A.T. Gomes, Waldir P. Elias

**Affiliations:** a Laboratório de Bacteriologia, Instituto Butantan, São Paulo, Brazil; b Institute of Hygiene, University of Münster, Münster, Germany; c Departamento de Pós-graduação, Faculdade de Medicina Veterinária, Universidade Santo Amaro, São Paulo, Brazil; d Instituto de Biociências, Universidade Estadual Paulista (UNESP), Botucatu, Brazil; e Departamento de Microbiologia, Imunologia e Parasitologia, Escola Paulista de Medicina, Universidade Federal de São Paulo, São Paulo, Brazil

**Keywords:** Hybrid-pathogenic*E. coli*, aggregate-forming pilus, AFP, urinary tract infection, intestinal colonization

## Abstract

Hybrid-pathogenic *Escherichia coli* represent an important group of strains associated with intestinal and extraintestinal infections. Recently, we described strain UPEC-46, a uropathogenic/enteroaggregative *E. coli* (UPEC/EAEC) strain presenting the aggregative adherence (AA) pattern on bladder and colorectal epithelial cells mediated by aggregate-forming pili (AFP). However, the role of AFP and other uninvestigated putative fimbriae operons in UPEC-46 pathogenesis remains unclear. Thus, this study evaluated the involvement of AFP and other adhesins in uropathogenicity and intestinal colonization using different *in vitro* and *in vivo* models. The strain UPEC-46 was able to adhere and invade intestinal and urinary cell lines. A library of transposon mutants also identified the involvement of type I fimbriae (TIF) in the adherence to HeLa cells, in addition to colorectal and bladder cell lines. The streptomycin-treated mouse *in vivo* model also showed an increased number of bacterial counts in the colon in the presence of AFP and TIF. In the mouse model of ascending urinary tract infection (UTI), AFP was more associated with kidney colonization, while TIF appears to mediate bladder colonization. Results observed in *in vivo* experiments were also confirmed by electron microscopy (EM) analyses. In summary, the *in vitro* and *in vivo* analyses show a synergistic role of AFP and TIF in the adherence and colonization of intestinal and urinary epithelia. Therefore, we propose that hybrid *E. coli* strains carrying AFP and TIF could potentially cause intestinal and urinary tract infections in the same patient.

## Introduction

Hybrid-pathogenic *Escherichia coli* comprises an important group of virulent strains, which harbour virulence characteristics of intestinal (IPEC) and extraintestinal pathogenic *E. coli* (ExPEC) [1]. One important member of this group includes the uropathogenic/enteroaggregative *E. coli* (UPEC/EAEC). These strains share some virulence traits of EAEC, such as the presence of EAEC-associated genes and/or the ability to produce the aggregative adherence (AA) pattern on epithelial cells [2–9], in addition to cause urinary tract infection (UTI). Recent epidemiological studies have associated the presence of these hybrid *E. coli* strains with its pathogenic potential [2–9]. However, some issues linked to genes involved in intestinal and urinary tracts colonization and their role in pathogenicity remain unclear.

In a recent study, we described the geno- and phenotypic characteristics of one *E. coli* strain (UPEC-46) isolated from a case of UTI [7]. This strain was classified as a hybrid-pathogenic UPEC/EAEC, as it was isolated from an extraintestinal infection, harboured EAEC virulence markers and was phylogenetically associated to EAEC strains [7]. Moreover, the aggregate-forming pili (AFP) were essential for establishing the AA pattern expressed by this strain in different cell lines [7].

AFP was initially described in a Shiga-toxigenic *E. coli* (STEC)/EAEC strain as a type IV pilus sharing a similar genetic organization with the bundle-forming pilus (BFP) present in enteropathogenic *E. coli* (EPEC) [10]. Following the first description, AFP-encoding genes were also found in EAEC strains isolated from healthy subjects and patients with diarrhea [7,11]. The ultrastructure of AFP was observed by transmission electron microscopy (TEM) using a specific anti-AFP serum, and it differed from BFP, presenting fimbrial structures not assembled in bundles [7]. AFP has also been associated with bacterial autoaggregation, adherence to bladder and colorectal epithelial cells and cytotoxicity towards epithelial cells in *stx*-positive *E. coli* [7,10]. However, the role of AFP in intestinal colonization and uropathogenesis remains unclear.

Thus, this study aimed to examine whether AFP and/or another adhesin present in the UPEC-46 strain is involved in intestinal colonization and uropathogenicity using different *in vitro* and *in vivo* experiments. A general analysis of virulence features of different adhesins is essential to understand their role in the pathogenesis of hybrid *E. coli* strains.

## Material and methods

### Bacterial strains, plasmids, and growth conditions

All bacterial strains and plasmids used in this study are described in Supplementary Table S1. The strains were grown in lysogeny broth (LB), LB agar, or MacConkey (MC) agar (Difco, USA) for 18 h at 37ºC and stored using LB supplemented with 15% (v/v) glycerol (Sigma-Aldrich, USA) at −80ºC. When necessary, the following antibiotics were added to the media: ampicillin (100 µg/mL), kanamycin (50 µg/mL), apramycin (100 µg/mL), chloramphenicol (25 µg/mL), or streptomycin (100 µg/mL) (Sigma-Aldrich).

### Adherence and invasion assays

Adherence assays were performed according to Schüroff et al. [7], using different human cells lines: cervical carcinoma (HeLa; ATCC CCL-2), colorectal adenocarcinoma (HT-29; ATCC HTB-38), and urinary bladder carcinoma (5637; ATCC HTB-9).

The assays were performed using Dulbecco modified Eagle medium (DMEM) (Cultilab, Brazil) for HeLa/HT-29 cells and Roswell Park Memorial Institute (RPMI) (Cultilab) for 5637 cells, supplemented with 2% (v/v) fetal bovine serum (Cultilab), and when necessary, 1% (w/v) D-mannose (Sigma-Aldrich).

The ability of bacteria to adhere to epithelial cell lines were assessed at a multiplicity of infection (MOI) of 1:100 with incubation of 3 h or 6 h at 37ºC under 5% CO_2_ and qualitative and quantitatively analyzed. For qualitative adherence assays, the preparations were fixed with methanol (Sigma-Aldrich), stained with May-Grunwald/Giemsa (Merck, Germany), and analyzed by light microscopy. For quantitative adherence assays, after interaction preparations were washed and lysed with 1% (v/v) Triton X-100 (Sigma-Aldrich). The bacteria counts were performed using MC agar plates.

For invasion assays, a quantitative bacterial infection of HeLa, HT-29, and 5637 cells was performed as described above, using incubation periods of 3 h. The cells were then washed with phosphate-buffered saline (PBS) and treated with 1 mL of respective culture medium (DMEM or RPMI) containing 100 µg/mL gentamicin (Sigma-Aldrich) for 1 h and lysed with 1% (v/v) Triton X-100 (Sigma-Aldrich). The bacteria counts were performed using MC agar plates. The invasion index was indicated as the ratio between internalized and total cell-associated bacteria expressed in percentage (%). All assays were performed three times in duplicate.

### Transposon mutants library

The EZ-Tn5 <R6K*γori*/KAN-2>Tnp Transposome kit (Epicentre, USA) was used for mutagenesis of UPEC-46 strain according to the manufacturer’s recommendations. Briefly, transformants were selected on LB agar containing kanamycin and analyzed by a 6-h adherence assay with HeLa cells in DMEM containing 1% D-mannose, following the methodology described above. Transposon mutants that lost the capacity to adhere in the AA pattern were selected for genomic DNA extraction and digestion with *EcoRI* (Thermo Fisher, USA), a restriction endonuclease that does not cleave the transposon sequence. The fragmented genomic DNA was self-ligated with T4 DNA ligase (Thermo Fisher) and inserted into *E. coli* DH5αλ*pir*. LB agar plates containing kanamycin were used for transformants selection. The plasmids obtained were extracted and integration sites of the transposons were confirmed by Sanger´s sequencing (ABI 3730 DNA Analyser System; Thermo Fisher) employing the primers described in Supplementary Table S2. The Basic Local Alignment Search Tool (BLAST - https://ncbi.nlm.nih.gov/genbank/) was used for sequence analyzes.

### Type I fimbriae mutation in UPEC-46

The strain UPEC-46:*afpA*, which is a previously described mutant carrying the suicide vector pJP5603, inserted into the *afpA* gene [7], was employed to generate a double mutant impaired in both AFP and type I fimbriae (TIF). For this purpose, *fimH*, which encodes the adhesive subunit of TIF, was mutated by the methodology of λRed recombination [12]. Briefly, the fragment containing the chloramphenicol resistance cassette was amplified by PCR from plasmid pKD3 with flanking regions matching the *fimH* gene, with the primers described in Supplementary Table S2. The PCR product was transformed into UPEC-46:*afpA* expressing the recombinases from plasmid pKOBEG-Apra. Transformants were selected on LB agar plates containing chloramphenicol and kanamycin. The pKOBEG-Apra plasmid was eliminated by multiple growth at 42 °C. The double mutant carrying the chloramphenicol resistance cassette, inserted into the *fimH* gene, was confirmed by Sanger sequencing, and named as UPEC-46:*afpA::fimH.*

The knockout of TIF in the selected double mutant (UPEC-46:*afpA::fimH*) was confirmed by agglutination assay on glass slides using mannan-rich yeast (*Saccharomyces cerevisiae*) cells [13]. UPEC-46:*afpA::fimH* was also phenotypically evaluated by means of growth curve and motility, as previously described [7].

### Mouse intestinal colonization model

The streptomycin-treated mouse model [14] was performed to investigate the capacity of UPEC-46 and derivative strains to colonize the intestinal tract. This experiment was performed following the protocol approved by the Ethics Committee on Animal Use of the Butantan Institute (Protocol # 7146050620). Seven-week-old female-specific pathogen-free (SPF) BALB/c mice were treated 48 h before inoculation and throughout the experiment with drinking water containing streptomycin (5 g/L). Before inoculation, SPF mice were orogastrically gavaged with a 0.4 M sodium bicarbonate solution. UPEC-46 and derivative strains were resuspended at a final concentration of 5.0 x 10^3^ colony forming units (CFU)/mL and 200 µL were administered by gavage. Fresh fecal samples were collected once a day until day 14 post-inoculation, weighted, diluted in PBS and plated onto MC agar plates containing the indicated antibiotic. The recovery of UPEC-46 or its derivatives on MC agar was confirmed by means of PCR amplification using specific primer (*afpA*, for UPEC-46, and *afpP*, for derivative strains) presented in Supplementary Table S2. The values obtained were expressed in CFU/g of feces. Groups of eight animals were tested for each strain. After euthanasia on day 14, intestinal fragments (duodenum, ileum, jejunum, and colon) were collected from one animal of each group of infected animals and processed for electron microscopy (EM) analyses. One animal inoculated with PBS (negative control) was also analyzed as described above and processed for EM analyses.

### UTI mouse model

A mouse model of ascending UTI was performed as previously described [15], with some modifications. This experiment was performed following the protocol approved by the Ethics Committee on Animal Use of the Butantan Institute (Protocol # 2178090819). Briefly, seven-week-old female SPF-C57BL/6 mice were anesthetized and 50 µL of bacterial suspension containing 1.0 x 10^9^ CFU was transurethrally inoculated. To evaluate the colonization ability of each strain, animals were sacrificed at different periods after inoculation: mice infected with UPEC-46 were evaluated after 6, 12, 24, or 72 h, while derivative strains were evaluated only after 72 h of infection. Immediately after euthanasia, the bladder and both kidneys were collected, weighed, and homogenized in 1 mL of PBS using the 20 mm stainless steel probe (G20) of the Omni Mixer homogenizer (Omni TH; Omni International Inc., Kennesaw, GA). Serial dilutions were plated onto MC agar containing the indicated antibiotic and the obtained values were expressed in CFU/g of organ. The recovery of UPEC-46 or its derivative constructions on MC agar was confirmed by means of PCR amplification using specific primers (*afpA*, for UPEC-46, and *afpP*, for derivative strains) presented in Supplementary Table S2. Groups of nine animals were inoculated with each strain. Organs (bladder and kidneys) collected from eight animals of each group were processed for CFU counting. Organs collected from the remaining infected animal of each group, and the negative control (one animal inoculated with PBS) wereanalyzed by EM.

### Electron microscopy

Tissue fragments obtained from *in vivo* experiments were primarily fixed in 2% glutaraldehyde (EMS, USA) and post-fixed with 1% osmium tetroxide (EMS) prepared with 0.1 M sodium cacodylate buffer. After fixation, specimens were dehydrated through a graded series of ethanol solutions (50%, 75%, 85%, 95%, and 100%) and propylene oxide (100%), and gradually embedded in Araldite resin. Ultrathin sections were placed onto 200 mesh copper grids previously coated with Formvar (EMS), and stained with 2% aqueous solution of uranyl acetate (Merck) and Reynold’s lead citrate (Merck). Grids were then examined under TEM (LEO 906E – Zeiss, Germany) [16].

For Scanning Electron Microscopy (SEM) analyses, tissue fragments were similarly fixed, post-fixed, and dehydrated as described for TEM. After dehydration with 100% ethanol, specimens were submitted to a critical point dry with carbon dioxide, and mounted onto SEM stubs. After receiving a thin layer of gold, specimens were examined under SEM (QUANTA 250 - FEI Company, Netherlands) at 12.5 kV [16].

### Statistical analyses

The GraphPad Software package (v7.0, GraphPad Software, USA) was used for statistical analyses. The Student’s *t*-test was employed to statistically compare UPEC-46 (wild-type) and derivative strains. Multiple comparisons were performed using the one-way analysis of variance (ANOVA) with *post hoc* Tukey HSD (honestly significant difference) test. The figures show the mean values ± standard deviations (SD) and *p* values <0.05 were considered statistically significant.

## Results

### UPEC-46 colonizes and invades intestinal and urinary tract cells in vitro

**Figure 1.**
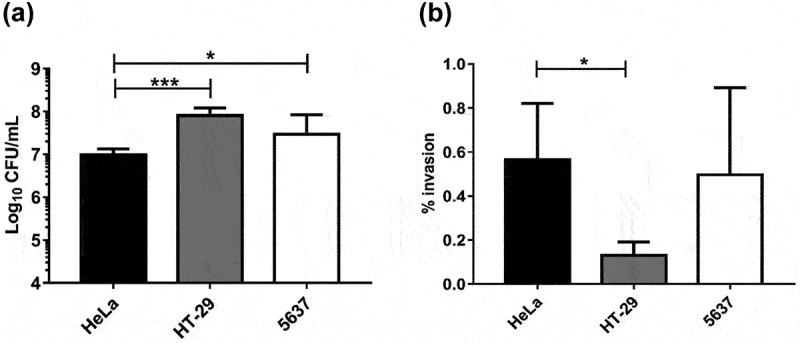
Adherence and invasion of UPEC-46 in different eukaryotic cell lines. (a) Quantitative adherence and (b) invasion assays for UPEC-46 were performed in a 3-h incubation period at 37 °C, without D-mannose, using HeLa, HT-29, or 5637 cells. Experiments were performed in biological triplicates and experimental duplicates. The ANOVA followed by Tukey’s multiple-comparison test was used for the statistical analyses. *P*-value:* *P* < 0.05; ***P < 0.001.

Initially, we evaluated the capacity of strain UPEC-46 to adhere to and invade cultivated eukaryotic cell lines from intestinal (HT-29) and urinary (5637) tracts origin. Analyzing the adherence rates of UPEC-46, we observed a higher number of adherent bacteria to HT-29 and 5637 cells, when compared to HeLa cells (*p* < 0.05; Figure 1(a)). In the invasion assay, UPEC-46 was able to invade all cell lines evaluated, with invasion rates not differing between HeLa and 5637 cells but significantly lower for HT-29 cells (Figure 1(b)), presenting invasion rates significantly lower than *S. flexneri* 2a strain (positive control) (*p* < 0.0001; Supplementary Figure S1).

### UPEC-46 can adhere to and colonize the bladder and kidneys in a UTI mouse model

**Figure 2.**
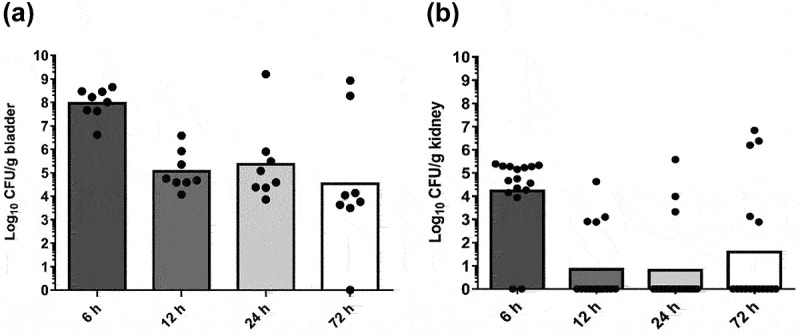
Colonization of bladders and kidneys mediated by UPEC-46 in a mouse model of ascending UTI. Groups of nine SPF-C57BL/6 mice were transurethrally inoculated with 1.0 x 10^9^ CFU of UPEC-46 strain and infections followed for 6, 12, 24, or 72 h. Animals were sacrificed and the (a) bladders (n = 8) and (b) kidneys (n = 16) from eight animals were collected for bacterial counts.

In view of results obtained *in vitro*, demonstrating the capacity of UPEC-46 to adhere and invade urinary tract epithelial cells, we also evaluated the ability of this strain to colonize the bladder and kidneys in an ascending UTI mouse model. Analyzing different post-inoculation times (6, 12, 24, and 72 h) by CFU counts, consistent bacterial colonization was observed in all periods, both in the bladder and kidneys (Figure 2). Therefore, considering the ability of UPEC-46 to colonize bladders and kidneys with high efficiency for up to 72 h, we selected this post-inoculation period for future analyses.

### AFP and TIF synergistically contribute to the establishment of the bacterial adherence

To identify other possible adhesins that could be involved in the adhesiveness of UPEC-46, we generated a transposon library from UPEC-46 strain using the EZ-Tn5 <R6K*γori*/KAN-2> transposon. A total of 1,040 transposon mutants were obtained and 12 presented considerably weak adherence and loss of the AA pattern feature in HeLa cells (Supplementary Figure S2). The analysis of flanking regions matching the transposon insertion led to the identification of different genes classified into three general categories: lipopolysaccharide (LPS) synthesis/modification, metabolism and cellular transport, and adhesins (Table 1). The adhesin-related genes were associated with the biogenesis of AFP and TIF.

**Table 1.** Insertion sites of EZ-Tn5 <R6K*γori*/KAN-2> transposon in UPEC-46 mutants displaying altered adherence on HeLa cells.

Mutant strain	Transposon insertion site	Gene category
204	*rfaG* – Glycosyltransferase	LPS synthesis/modification
310	*yahK* – Aldehyde reductase	Metabolism and cell transporter
316	*afpP -* Prepilin peptidase	AFP adhesin
441	*fimI -* FimI protein	TIF adhesin
632	*afpB -* Membrane protein (secretin)	AFP adhesin
700	*afpE -* Inner membrane protein	AFP adhesin
780	*pyrB* - Aspartate carbamoyltransferase	Metabolism and cell transporter
781	*afpB -* Membrane protein (secretin)	AFP adhesin
854	*nhaA* - Na(+)/H(+) antiporter	Metabolism and cell transporter
901	*afpD -* DNA binding protein	AFP adhesin
936	*rfaL* – O-antigen ligase	LPS synthesis/modification
976	*pfkL* − 6-phosphofructokinase	Metabolism and cell transporter

**Figure 3.**
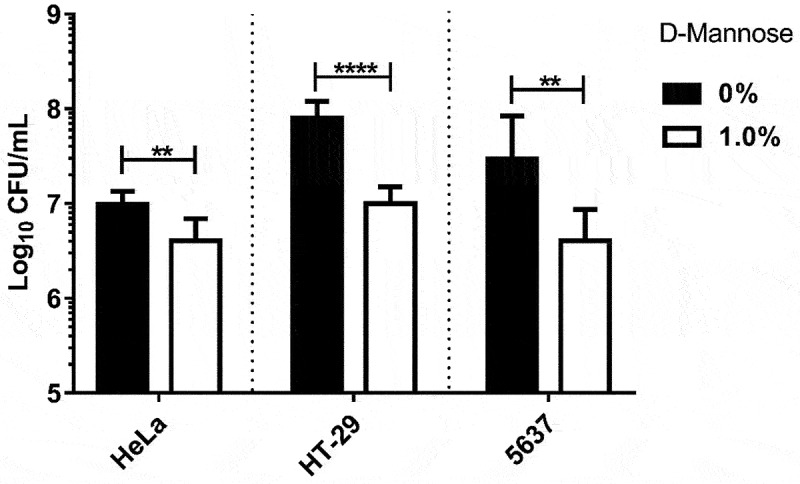
D-Mannose inhibition of adherence of UPEC-46 on different eukaryotic epithelial cell lines. UPEC-46 was submitted to quantitative adherence assays (incubation period of 3 h at 37°C) using HeLa, HT-29, or 5637 cell lines. Adherence of UPEC-46 to different cells was quantified in the absence or presence of 1% D-mannose. The experiments were performed in biological triplicates and experimental duplicates. The Student’s *t-* test was used for the statistical analyses. *P*-value: **P < 0.01; ****P < 0.0001.

TIF have been repeatedly associated with adherence in the urinary tract, contributing to the pathogenesis of UPEC strains [17,18]. Thus, we investigated the contribution of this adhesin to the adherence of UPEC-46. Initially, a quantitative adherence assay was performed in different cell lines in the absence or presence of 1% D-mannose. A significantly lower number of adherent bacteria was observed when 1% D-mannose was used (*p* < 0.01; Figure 3), indicating that TIF also contributed to the adherence of UPEC-46.

TIF mutation was obtained using the UPEC-46:*afpA* mutant to confirm its role in UPEC-46 adherence. The UPEC-46:*afpA::fimH* mutant was initially evaluated by different phenotypic tests. No differences were observed in growth rate and motility when UPEC-46 and UPEC-46:*afpA::fimH* were compared. Also, yeast agglutination was not observed in the UPEC-46:*afpA::fimH* in the absence of D-mannose, confirming that TIF production was abolished in this strain (Supplementary Figure S3).

**Figure 4.**
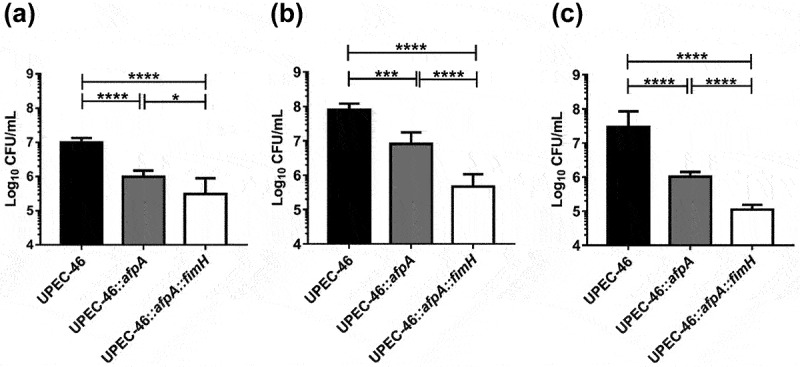
Role of AFP and TIF in the adherence of UPEC-46 on different eukaryotic epithelial cell lines. Quantitative adherence assays for UPEC-46, UPEC-46:*afpA*, and UPEC-46:*afpA:fimH* were performed in a 3-h incubation period at 37°C, without D-mannose, using (a) HeLa, (b) HT-29, or (c) 5637 cell lines. Experiments were performed in biological triplicates and experimental duplicates. The ANOVA followed by Tukey’s multiple-comparison test was used for the statistical analyses. *P*-value:*P < 0.05; ***P < 0.001; ****P < 0.0001.

Additionally, adherence assays for UPEC-46, UPEC-46:*afpA*, and UPEC-46:*afpA::fimH* were performed using HeLa, HT-29, and 5637 cells. Qualitative adherence assays showed that the AA pattern was considerably impaired in UPEC-46:*afpA* and almost completely abolished for UPEC-46:*afpA::fimH* in all lines analyzed (Supplementary Figure S4). In the quantitative adherence assay, UPEC-46:*afpA::fimH* exhibited lower adherence to all cells when compared to UPEC-46:*afpA* (*p* < 0.05; Figure 4). Thus, results show that as well as AFP, TIF also contribute to the adhesiveness of UPEC-46.

### AFP and TIF promote intestinal colonization of UPEC-46 in a mouse model

**Figure 5.**
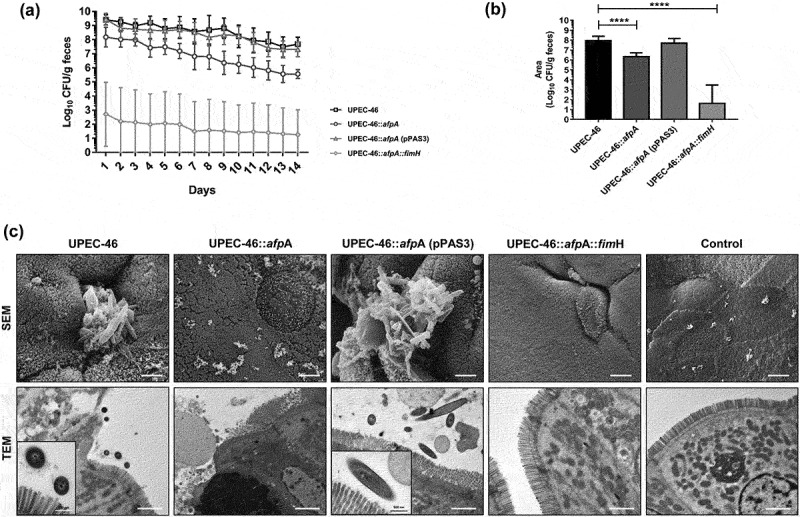
Role of AFP and TIF in the intestinal colonization in streptomycin-treated mouse model. Groups of eight SPF-BALB/c mice were orogastrically inoculated with 1.0 x 10^3^ CFU of UPEC-46 and derivative strains. Fresh fecal samples were collected once a day from each mouse for up to 14 days post-inoculation for (a) bacterial counts and obtaining the (b) area under the curve (cumulative bacterial counts) expressed in CFU/g of fecal. (c) SEM and TEM of colon fragments collected from mice infected with UPEC-46 and derivative strains on day 14. Non-infected colon fragment was used as a negative control. Bars, 2 µm. The Student’s *t*-test was used for the statistical analyses, comparing UPEC-46 and derivative strains. *P*-value: ****P < 0.0001.

UPEC-46 colonized the intestinal epithelia of mice up to day 14 post-inoculation, with values ranging from 10^7^ to 10^10^ CFU/g feces. UPEC-46:*afpA* showed lower level of colonization and cumulative bacterial counts during the 14 days analyzed (*p* < 0.0001; Figure 5(a,b)). Concomitantly, the double mutant UPEC-46:*afpA::fimH* showed the highest reduction in intestinal colonization levels. While colonization in some mice was around 10^4^ CFU/g feces for the first few days after inoculation, bacterial counts gradually declined until they were no longer detected. Detectable colonization with UPEC-46:*afpA::fimH* was not observed in four mice during the whole period of experimentation (Supplementary Figure S5). Complemented UPEC-46:*afpA* (pPAS3) restored intestinal colonization levels with similar cumulative bacterial counts when compared to UPEC-46 (Figure 5(a,b)).

Intestinal fragments (duodenum, jejunum, ileum, and colon) were also analyzed by EM. Bacterial adherence to the epithelium was not observed in the duodenum and jejunum. In the ileum, bacterial adherence was mainly observed in animals infected with UPEC-46:*afpA* (pPAS3) (Supplementary Figure S6). In the colon, the number of adherent bacteria observed was considerably higher. Bacterial adherence on the colon surface was observed in mice infected with UPEC-46, but not in mice infected with UPEC-46:*afpA* or UPEC-46:*afpA::fimH* mutants. UPEC-46:*afpA* (pPAS3) not only completely restored, but also increased the capacity of the complemented strain to adhere to the epithelial cells in the colon (Figure 5(c)). No adherent bacteria or histological changes were observed in the intestinal fragments of the control animal inoculated with PBS.

### AFP and TIF promote UPEC-46 colonization in mice urinary tract

**Figure 6.**
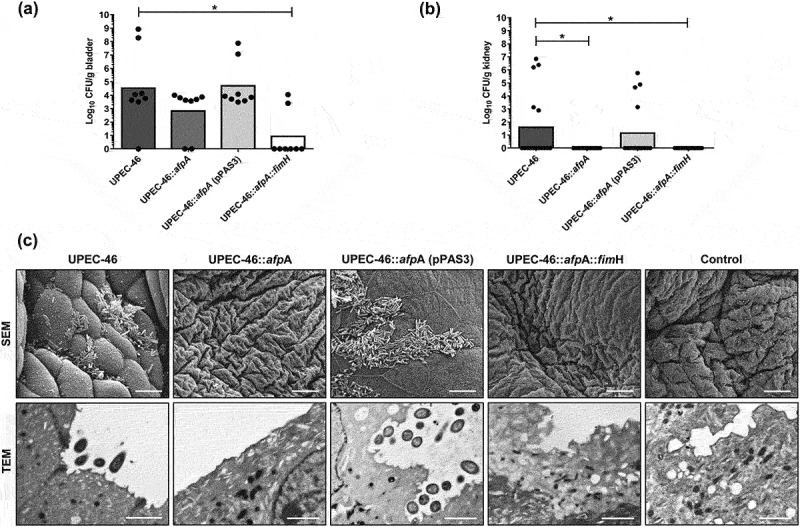
Role of AFP and TIF in the colonization of bladders and kidneys in a mouse model of ascending UTI. Groups of nine SPF-C57BL/6 mice were transurethrally inoculated with 1.0 x 10^9^ CFU of UPEC-46 and derivative strains. Three days after inoculation, mice were sacrificed and the (a) bladders (n = 8) and (b) kidneys (n = 16) from eight animals were collected for bacterial counts. (c) SEM and TEM of bladder fragments obtained from mice infected with UPEC-46 and derivative strains, 72 h post-inoculation. Non-infected bladder fragments were used as negative controls. Bars: SEM, 10 µm and TEM, 2 µm. The Student’s *t-* test was used for the statistical analysis, comparing UPEC-46 and derivative strains. *P*-value:*P < 0.05.

UPEC-46 colonized the bladder and kidneys, presenting bacterial counts of approximately 10^5^ CFU/g and 10^2^ CFU/g, respectively (Figure 6(a,b)). Mutation in *afpA* reduced the bacterial counts in the bladder, but only the double mutation present in UPEC-46:*afpA::fimH* significantly reduced the bladder colonization index (*p* < 0.05). Regarding kidney colonization, it was observed that UPEC-46:*afpA* was not able to colonize this organ, evidencing an important role of AFP in kidney colonization (Figure 6(b)). In addition, UPEC-46:*afpA* (pPAS3) strain restored colonization levels in bladders and kidneys similar to UPEC-46.

Bladder and kidney fragments of UPEC-46 and derivative strains were also analyzed by SEM and TEM. Bacterial adherence to the bladder cells’ surface was observed in mice infected by UPEC-46, but not in mice infected with the mutants UPEC-46:*afpA* or UPEC-46:*afpA::fimH* (Figure 6(c)). UPEC-46:*afpA* (pPAS3) restored the adherence in the bladder. UPEC-46 adherence to kidney tissue was not observed by SEM. However, some cellular disorganization and partial detachment of the kidney epithelial cells were present in preparations infected by UPEC-46 and complemented strain UPEC-46:*afpA*, but not in the *afpA* or *afpA*/*fimH* mutants (Supplementary Figure S7).

## Discussion

Hybrid-pathogenic UPEC/EAEC strains have been recently described as important bacterial infectious agents, isolated from cases of diarrhea and/or UTI [2–9,15]. Despite this emerging epidemiological importance, some aspects related to the pathogenesis of these strains still need to be better investigated.

Adherence and invasion to host cells are important virulence mechanisms of pathogenic *E. coli* [19–21]. Recent studies have described UPEC/EAEC strains adhering to intestinal and urinary tract epithelial cells, suggesting their virulence potential for intestinal and extraintestinal infections [5,7,9]. In fact, in our study we confirmed that UPEC-46 is able to adhere on cultivated HT-29 and 5637 cell lines, by means of quantitative adherence assays. Additionally, UPEC-46 was also able to invade the same epithelial cell lines. Thus, these findings suggest that UPEC-46 could be associated with infection and colonization of intestinal and urinary tracts.

In our previous study, we demonstrated that AFP was associated with the adhesiveness of UPEC-46 on intestinal and urinary cell lines, and the AFP mutant strain (UPEC-46:*afpA*) was still able to adhere weakly, suggesting that other redundant adhesins could be expressed [7]. In fact, it was found that the genome of UPEC-46 contains several other uninvestigated putative adhesin operons [7]. By analyzing different transposon mutants, we confirmed the role of AFP in UPEC-46 adhesiveness and identified the involvement of TIF and some metabolism-related enzymes in the colonization process. Six transposon insertions disrupted genes involved in cellular physiology, such as LPS biosynthesis, ion transport, and metabolic pathways. Some studies have shown that mutation in genes involved in these cellular processes indirectly affects adherence, biofilm formation, and motility of different Gram-negative bacteria [22–25].

The role of TIF in establishing the AA pattern has also been demonstrated in EAEC prototype strain 042 [26]. In our study, adherence assays with 1% D-mannose significantly decreased the adherence of UPEC-46 strain, indicating a role of TIF in this phenotype. To further investigate the role of AFP and TIF in adherence, we constructed a mutant strain in both adhesins. The constructs previously obtained concerning the *afpA* gene [7] were also used for subsequent analyses. Adherence assays showed that AFP works in synchronicity with TIF in establishing UPEC-46 adherence. Indeed, some studies have shown that different fimbrial operons can act in synergy, facilitating the colonization and contributing to the pathogenesis in the host [27,28]. Therefore, *in vivo* studies became necessary to evaluate the role of AFP and TIF in the colonization of urinary and intestinal tracts.

UPEC-46 efficiently colonized the intestinal tract of mice, similarly to the EAEC prototype strain 042 [14] and the atypical enteropathogenic *E. coli* BA589 [29] that also produces the AA pattern. Analyzing the colonization kinetics in the intestinal tract, we showed that UPEC-46:*afpA* caused a significant decrease in the cumulative bacterial counts compared to UPEC-46. This result indicates that AFP has a role in intestinal colonization, with this study being the first to show the importance of this adhesin in the pathogenesis of *E. coli* causing intestinal infections. Although the murine intestinal colonization model employed in our study is not considered appropriate to evaluate diarrhea [14], two AFP-positive strains (STEC/EAEC 12–05829 and EAEC 12–05898) described by Lang et al. [10], and several other EAEC AFP-positive isolates [7,11], were isolated from diarrhea cases, indicating the diarrheagenic potential of AFP-positive strains. In addition, our results also showed that inactivation of both AFP and TIF led to a remarkable decrease in the establishment of intestinal colonization, throughout the entire experiment. Other studies have demonstrated the role of TIF in *E. coli* intestinal colonization using the same model [30–32]. Thus, our results for UPEC-46 and its derivatives indicate a role of AFP and TIF in intestinal colonization.

Analysis of different portions of the intestines of the colonized mice showed that UPEC-46 could colonize the distal parts of the intestinal tract, mainly the colon. EAEC and other pathogenic *E. coli* strains have been mainly associated to colon colonization [33,34]. The presence in these specific site could facilitate UPEC-46 interactions with other intestinal pathogens, allowing the sharing of characteristics associated with metabolism and virulence [35]. The fact that mutant strains (UPEC:*afpA* and UPEC:*afpA::fimH*) were unable to colonize the mouse colon also confirms the importance of AFP and TIF in the intestinal colonization.

The mouse model of ascending UTI has been used to characterize the uropathogenic potential of different *E. coli* strains [15,22,36]. In the kinetic of bladder and kidney colonization, UPEC-46 presented uropathogenic traits associated with the UPEC pathotype [37], demonstrating the ability to persist in the bladders of infected mice for periods up to 72 h post-inoculation. Analyzing the role of AFP in bladder and kidney colonization, we observed that this adhesin is important to kidney colonization, whereas there were no significant differences in bladder colonization. Also, this is the first study showing the association of AFP with urovirulence. In addition, the *fimH* mutation, present in UPEC:*afpA::fimH*, significantly reduced bladder colonization in mice. These data corroborate the results previously described [15,32,36], where TIF appears to mediate bladder colonization of *E. coli* strains.

EM analyses confirmed the relationship between adherence and TIF in the bladder. However, no adherence of UPEC-46 was observed in kidneys by SEM. We hypothesized that our inability to detect bacteria in this assay could be attributed to the low bacterial counts in the kidneys and/or to potential bacterial loss during the fragments´ processing. More extended infection periods could also be useful to study bacterial adherence/colonization in kidneys by SEM.

In summary, *in vitro* and *in vivo* analyses performed in this study suggest that AFP and TIF play an essential role in the pathogenesis of the hybrid-pathogenic UPEC/EAEC strain UPEC-46, mediating bacterial adherence and urinary tract and intestinal epithelial colonization. Bacterial interaction with these epithelia could result in bacterial spread into the bloodstream. In fact, virulence factors-encoding genes related to serum resistance, such as *iss* (increased serum survival) and group IV capsule [38,39], were present in the UPEC-46 genome, indicating its potential to survive in the bloodstream [7]. Finally, our results may not represent a general characteristic of UPEC/EAEC strains, once hybrid *E. coli* strains may present different genetic backgrounds, but they do suggest that AFP and TIF may play an essential role in their pathogenesis, allowing these strains to colonize both the urinary and the intestinal tracts.

## Supplementary Material

Supplemental Material

## Data Availability

The authors confirm that the data supporting the findings of this study are available within the article and its supplementary materials. The raw data used in our analyses are available in the Butantan Institute Repository [https://repositorio.butantan.gov.br/handle/butantan/4315].
