# Supplementary material for: Role of aggregate-forming pilus (AFP) in adherence and colonization of both intestinal and urinary tracts
Source: Virulence. 2022 Aug 18;13(1):1423–33. doi: 10.1080/21505594.2022.2112818 (PMC9397481; doi:10.1080/21505594.2022.2112818)
Supplement: Supplemental Material [file KVIR_A_2112818_SM5228.pdf]

Supplementary Material

Supplementary Table 1. Bacterial strains and plasmids used in this study.

	Characteristics	References
**Strains**		
UPEC-46	*E. coli* wild-type strain, O166:H12	[1-3]
UPEC-46::*afpA*	UPEC-46 *afpA* mutant	[3]
UPEC-46::*afpA* (pPAS3)	UPEC-46::*afpA* strain complemented with *afpA* gene	[3]
UPEC-46::*afpA::fimH*	UPEC-46 *afpA-fimH* double mutant	This study
E. coli DH5α	K-12 *E. coli* strain for plasmid propagation	Stratagene
E. coli DH5α(λ*pir*)	K-12 *E. coli* strain lysogenized with lambda phage and harbouring the *pir* gene	[4]
E. coli C600	*E. coli* strain used as a negative control for invasion test	[5]
S. flexneri 2a	*Shigella flexneri* strain used as a positive control for invasion and negative control for motility assays	[6]
**Plasmids**		
pKOBEG-Apra	Derivative of pKOBEG plasmid carrying the λ phage red operon	[7]
pKD3	Lambda Red template plasmid	[8]

Supplementary Table 2. Primer sequences, amplified product sizes, and annealing temperatures used in this study.

Primers	Sequence (5'-3')	Annealing temp (°C)	Size of PCR product (bp)	References
***afpA* gene amplification**				
FwAfpA	AATGCTCGAGATGAATATTTTTACAAAAAAAG	56	635	[3]
RvAfpA	TCACAAGCTTTTATTTCAGCAGGAAGGT			
***afpP* gene amplification**				
FwAfpP	AATGTCCTGAGTGTGGAGTG	56	478	This study
RvAfpP	ATAGGAGCAAAGCGAATGCC			
**Nonpolar mutant construction (Lambda Red)**				
FwRedFimH	ATGAAACGAGTTATTACCCTGTTTGCTGTACTGCT GATGGGCTGGTCGGTAAATGCCTGGTCATTCGCC TGTAAAACCTGTGTAGGCTGGAGCTGCTTCG	50	1,175	This study
RvRedFimH	TTATTGATAAACAAAAGTCACGCCAATAATCGAT TGCACATTCCCTGCAGTCACCTGCCCTCCGGTAC GTGCGTAATTTGCATATGAATATCCTCCTTAG			
**Confirmation of mutation constructed by the Lambda Red system**				
FimH-Fw	CTCACAATCAGCGCACTTCC	56	1,122	This study
FimH-Rv	CGCGTCTTATCTGGCCTACA			
**Sequencing of the EZ-Tn5 transposon insertion site**				
KAN-2 FP-1	ACCTACAACAAAGCTCTCATCAACC	-	-	Epicentre
R6KAN-2 RP-1	CTACCCTGTGGAACACCTACATCT			

References

1. Abe CM, Salvador FA, Falsetti IN, et al. Uropathogenic *Escherichia coli* (UPEC) strains may carry virulence properties of diarrhoeagenic *E. coli*. FEMS Immunol Med Microbiol. 2008;52(3):397-406.
2. Nunes KO, Santos ACP, Bando SY, et al. Enteroaggregative *Escherichia coli* with uropathogenic characteristics are present in feces of diarrheic and healthy children. Pathog Dis. 2017;75(8):10.1093/femspd/ftx106.
3. Schüroff PA, Salvador FA, Abe CM, et al. The aggregate-forming pili (AFP) mediates the aggregative adherence of a hybrid-pathogenic *Escherichia coli* (UPEC/EAEC) isolated from a urinary tract infection. Virulence. 2021;12(1):3073-3093.
4. Elliott SJ, Kaper JB. Role of type 1 fimbriae in EPEC infections. Microb Pathog. 1997;23(2):113-118.
5. Appleyard RK. Segregation of new lysogenic types during growth of a doubly lysogenic strain derived from *Escherichia coli* K12. Genetics. 1954;39(4):440-452.
6. Bârzu S, Benjelloun-Touimi Z, Phalipon A, et al. Functional analysis of the *Shigella flexneri* IpaC invasin by insertional mutagenesis. Infect Immun. 1997;65(5):1599-1605.
7. Sampaio SC, Gomes TA, Pichon C, et al. The flagella of an atypical enteropathogenic *Escherichia coli* strain are required for efficient interaction with and stimulation of interleukin-8 production by enterocytes *in vitro*. Infect Immun. 2009;77(10):4406-4413.
8. Datsenko KA, Wanner BL. One-step inactivation of chromosomal genes in *Escherichia coli* K-12 using PCR products. Proc Natl Acad Sci U S A. 2000;97(12):6640-6645.

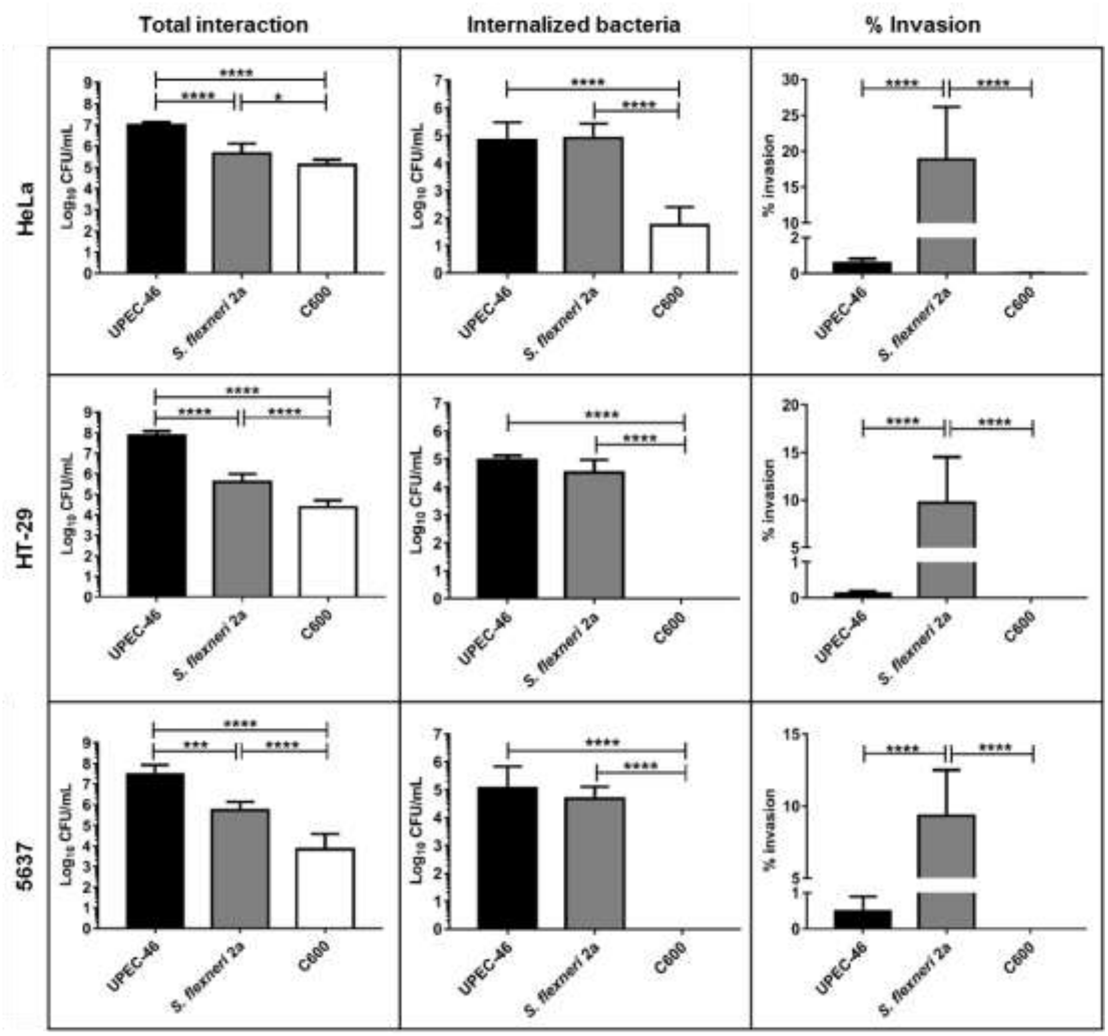

Supplementary Figure 1. Adherence and invasion of UPEC-46 in different eukaryotic cell lines. Assays were performed in a 3-h incubation period, without D-mannose, using **(a)** HeLa (human cervical carcinoma), **(b)** HT-29 (human colorectal adenocarcinoma), or **(c)** 5637 (human urinary bladder carcinoma) cells. Total numbers of adhered and internalized bacteria were evaluated for UPEC-46, invasive *S. flexneri* 2a, and non-invasive *E. coli* C600, used as control strains. The invasion index was indicated as the ratio between internalized and total cell-associated bacteria expressed as a percentage (%). The experiments were performed in biological triplicates and experimental duplicates. The ANOVA followed by Tukey's multiple-comparison test was used for the statistical analysis. *P*-values: * P < 0.05; *** P < 0.001; **** P < 0.0001.

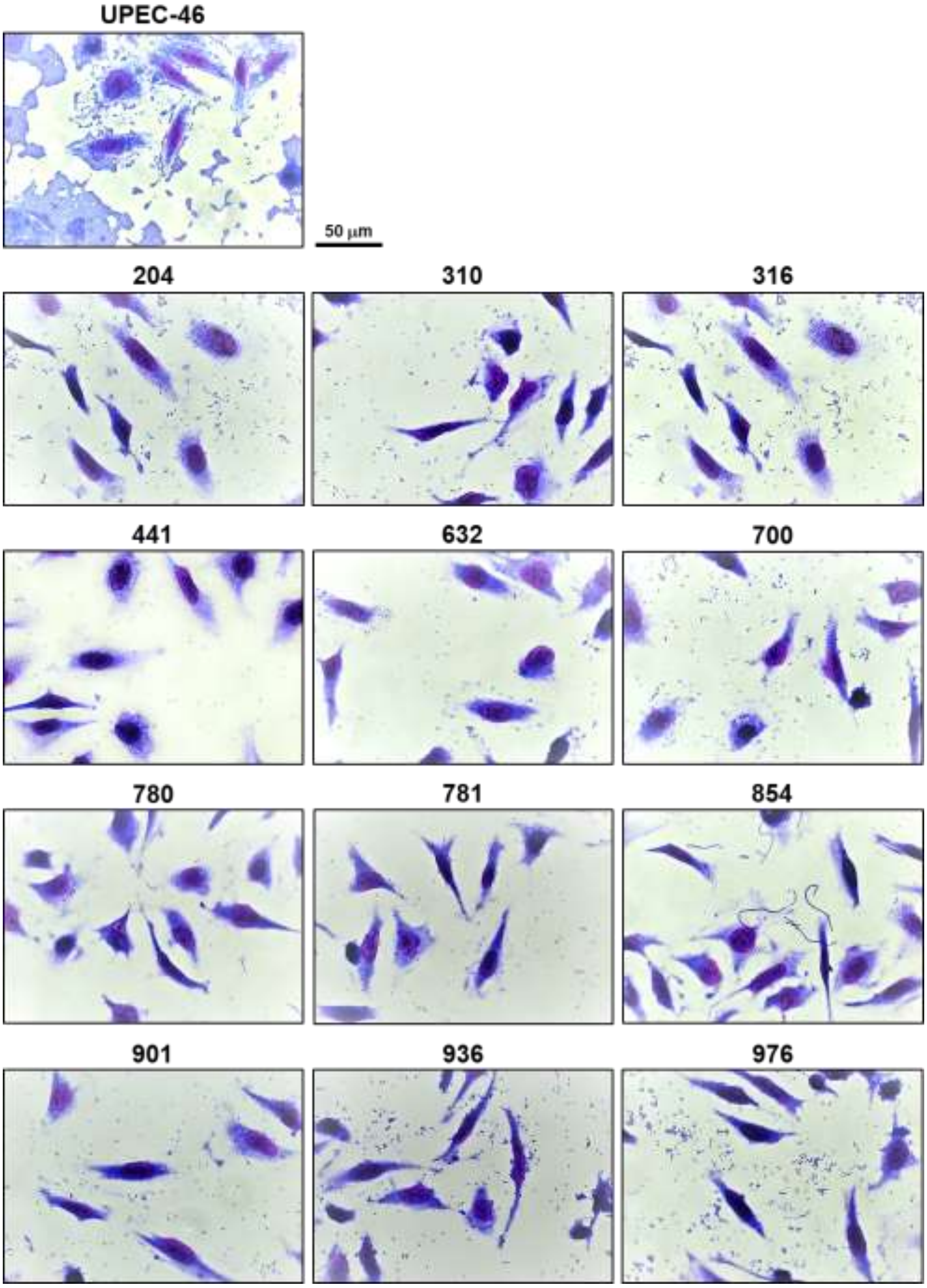

Supplementary Figure 2. Adherence characteristics of mutants selected from the library of UPEC-46 transposon mutagenesis. Qualitative 6 h-adherence assay on HeLa cells of select mutants that no longer presented the AA pattern. The assays were performed in the presence of 1% D-mannose. Bacterial adherence was evaluated by light microscopy after staining with May-Grunwald/Giemsa. UPEC-46 is depicted as the control for the AA pattern. Bars = 50 µm.

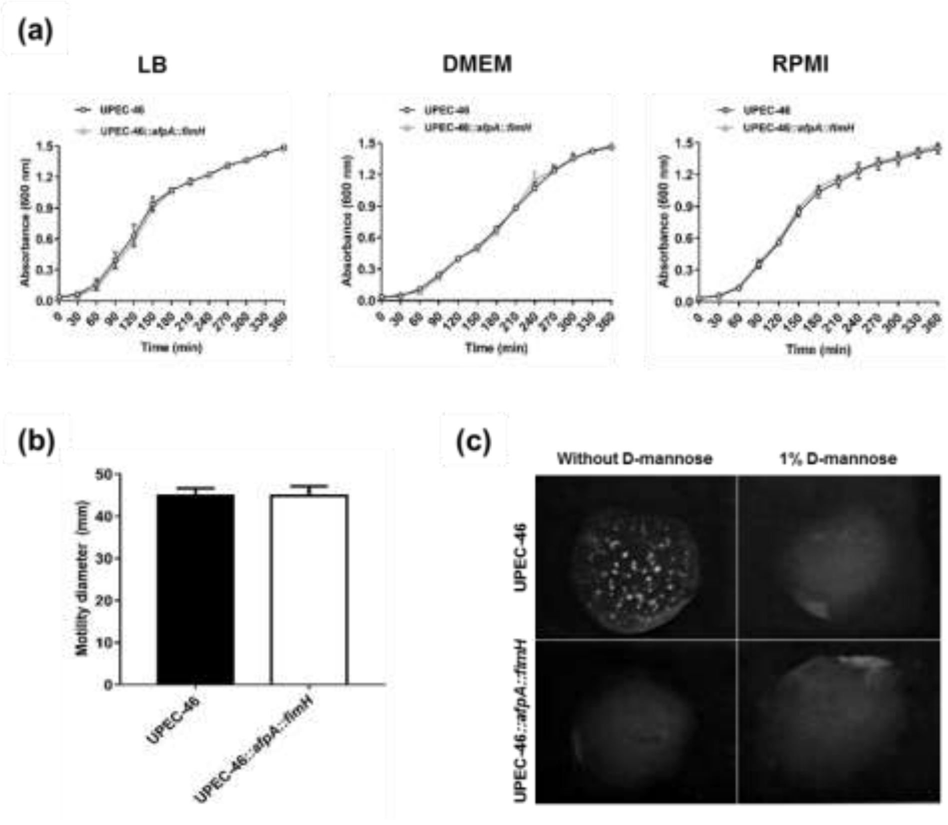

Supplementary Figure 3. Phenotypic characteristics of UPEC-46*::afpA::fimH*. (a) Growth curve of UPEC-46 and UPEC-46::*afpA*::*fimH,* using lysogeny broth (LB), Dulbecco modified Eagle medium (DMEM), and Roswell Park Memorial Institute medium (RPMI). Each culture was incubated at 37 °C with shaking (250 rpm). Absorbance was measured every 30 min for 6 h at 600 nm. The experiments were performed in biological triplicates. **(b)** Motility of UPEC-46 and UPEC-46::*afpA*::*fimH* in motility agar (LB and agar 0.3%) at 37 ºC during 18 h. *S. flexneri* 2a was used as a negative control and experiments were performed in biological triplicates and experimental duplicates. The Student's *t*-test was used for the statistical analysis, comparing UPEC-46 and UPEC-46::*afpA*::*fimH.* **(c)** Surface-expressed type I fimbriae (TIF) on UPEC-46 and UPEC-46::*afpA*::*fimH* strains were assayed employing the agglutination of yeast cells (*Saccharomyces cerevisiae*) on glass slides. Equal volumes of bacterial and yeast suspensions were mixed on a glass slide and rocked for 1 to 2 min. Bacterial aggregates were macroscopically analyzed. To confirm that TIF-mediated yeast agglutination was due to the specific recognition of mannosidic residues, the assays were also performed in the presence of 1% D-mannose.

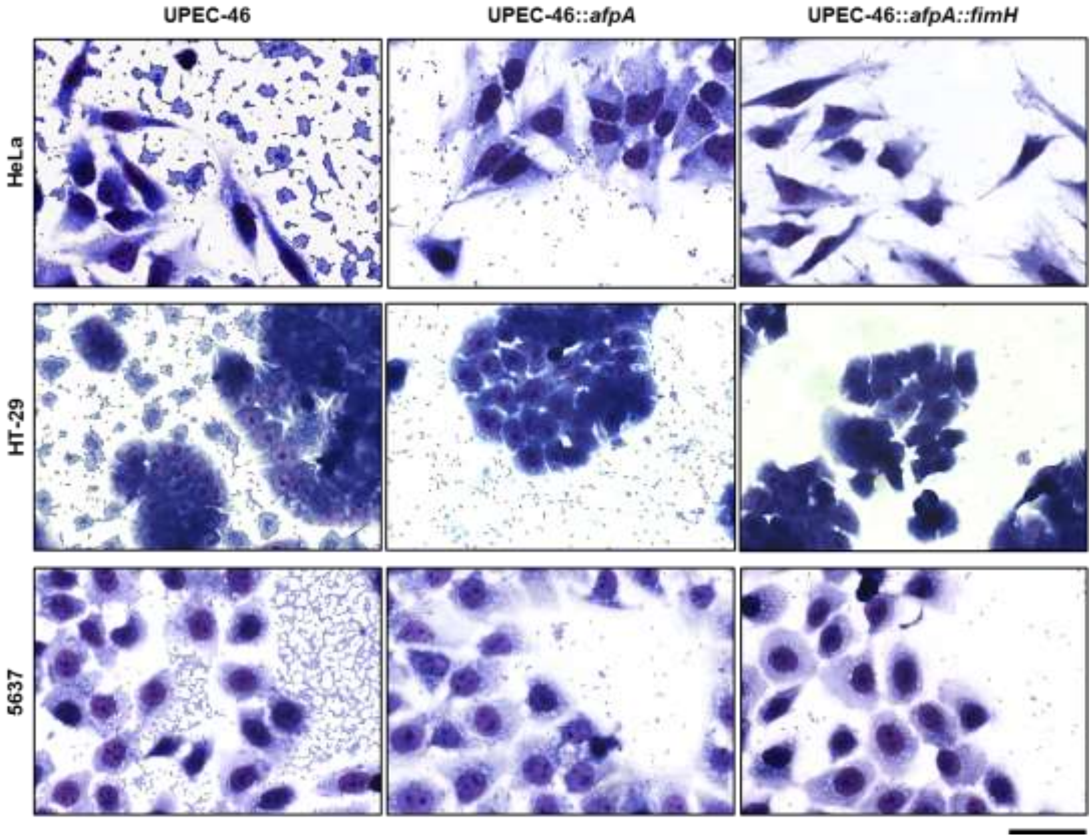

Supplementary Figure 4. Role of AFP and TIF on adherence characteristics of UPEC-46 strain in different eukaryotic cell lines. Qualitative adherence assays with UPEC-46, UPEC-46::*afpA*, or UPEC-46::*afpA*::*fimH* were performed in 3-h assays without D-mannose using HeLa, HT-29 or 5637 cells. Bacterial adherence was evaluated by light microscopy after staining with May-Grunwald/Giemsa. Bars = 50 µm.

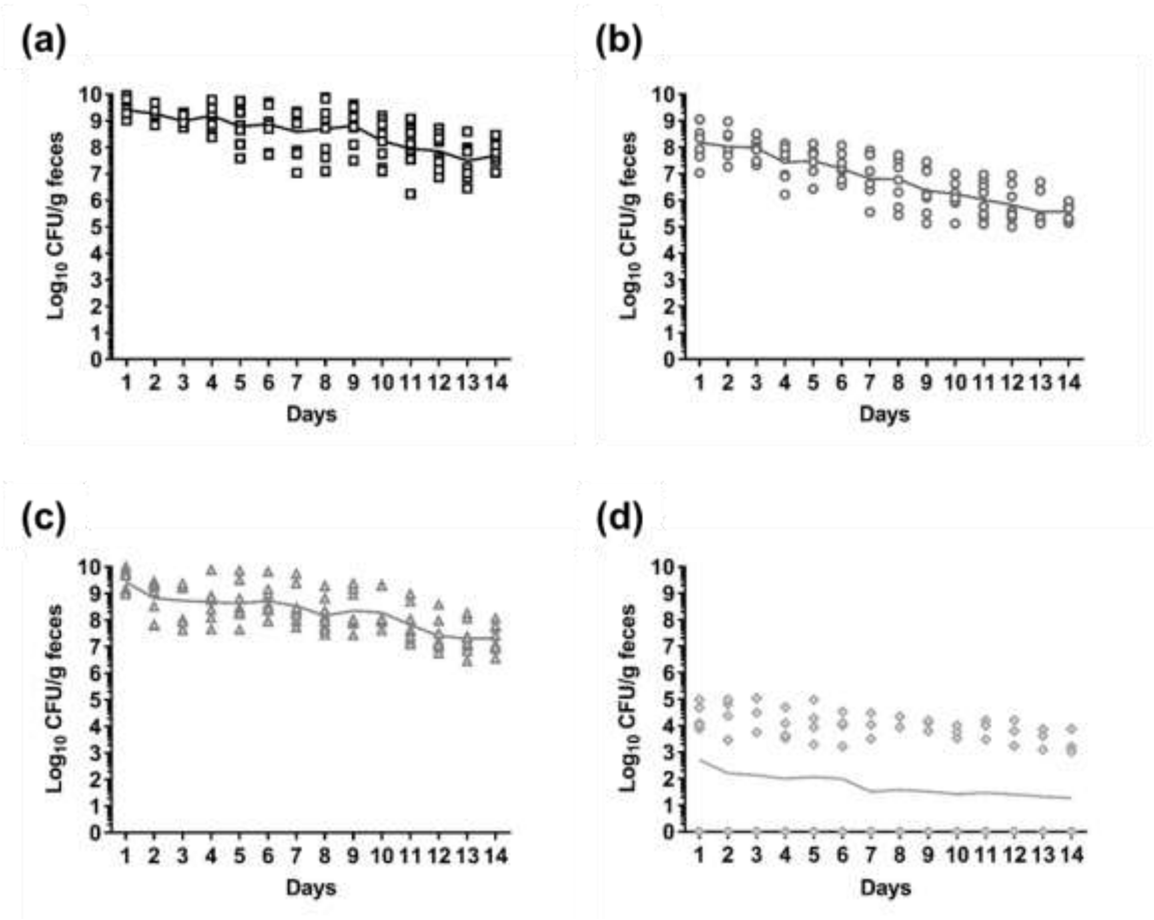

Supplementary Figure 5. Individual intestinal colonization values obtained for each mouse evaluated in the streptomycin-treated mice model. Groups of eight SPF-BALB/c mice were orogastrically inoculated with 1.0 x 10^3 CFU of **(a)** UPEC-46, **(b)** UPEC-46::*afpA*, **(c)** UPEC-46::*afpA* (pPAS3), and **(d)** UPEC-46::*afpA*::*fimH*. Fresh fecal pellets were collected for bacterial counts from each mouse for up to 14 days post-inoculation. Individual values for each mouse are represented by different symbols and the line represents the medium value expressed in Log_{10} CFU/g feces.

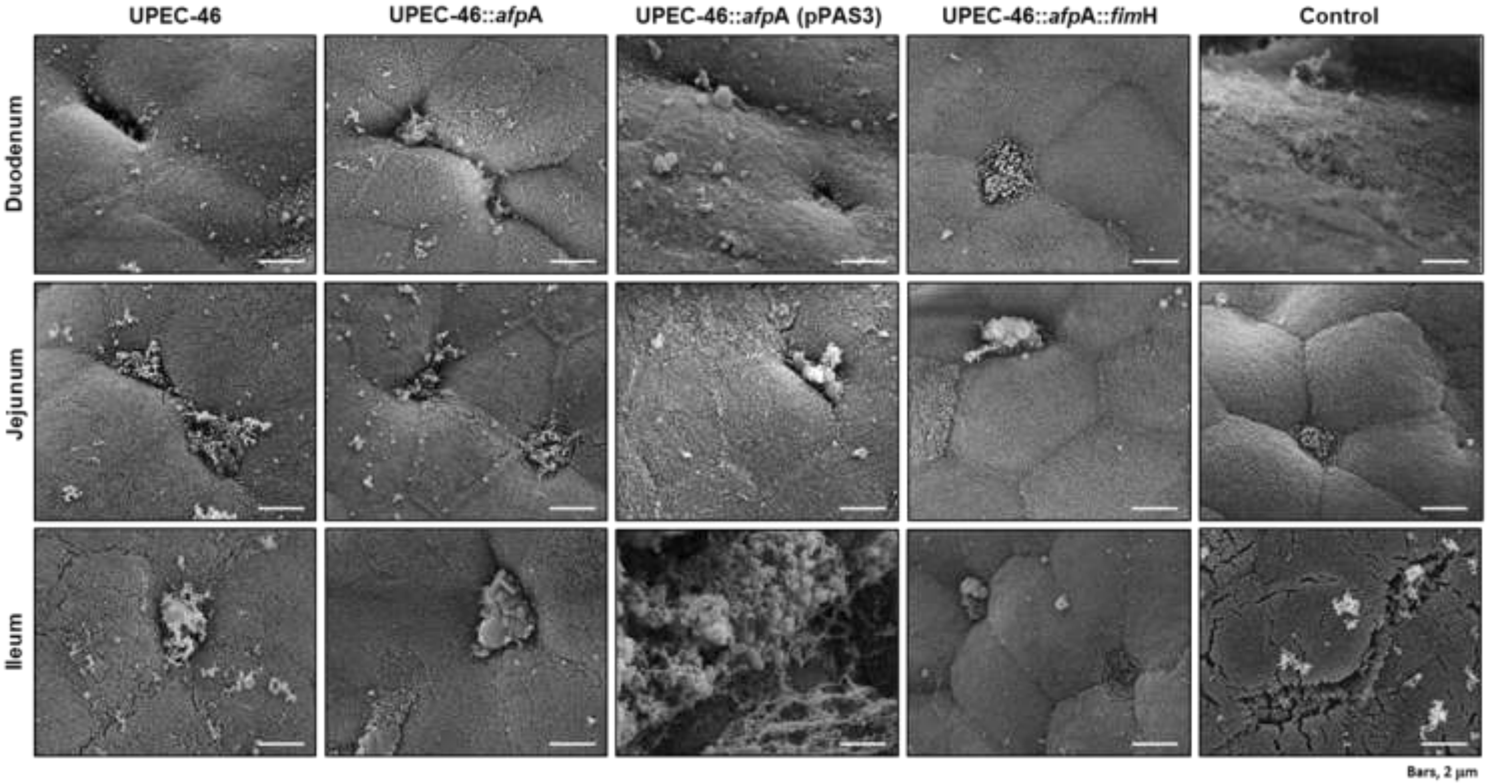

Supplementary Figure 6. Evaluation of intestinal colonization on duodenum, jejunum, and ileum in streptomycin-treated mouse model. Initially, SPF-BALB/c mice were orogastrically inoculated with 1.0 x 10^3 CFU of UPEC-46 and derivative strains. Fragments from different intestinal portions (duodenum, jejunum, or ileum) were collected 14 days post-inoculation and analyzed by SEM. Fragments of organs collected from an animal inoculated with PBS were used as negative controls. Except for the ileum fragment infected with UPEC-46*::afpA* (pPAS3) strain, bacterial adherence were not observed in other fragments.

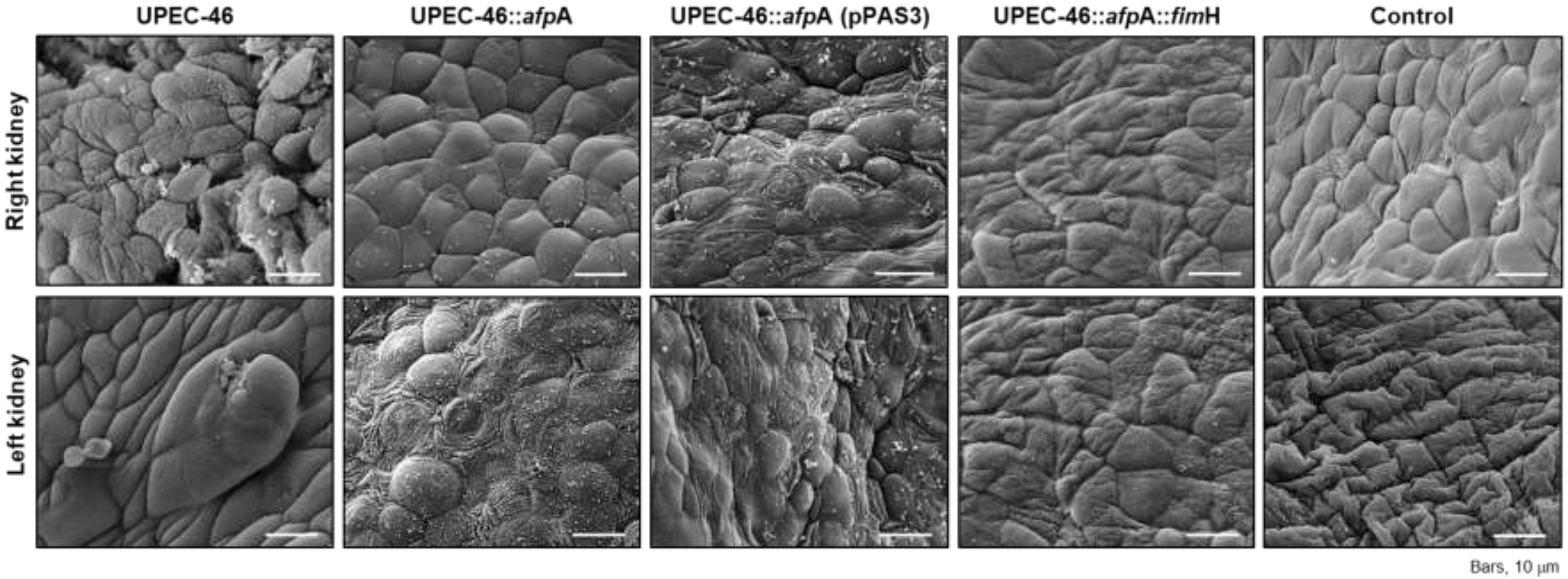

Supplementary Figure 7. Evaluation of kidney colonization in a mouse model of ascending UTI. SPF-C57BL/6 mice were transurethrally inoculated with 1.0 x 10^9 CFU of UPEC-46 and derivative strains. Three days after inoculation, mice were euthanized, kidneys collected and analyzed by SEM. A non-infected kidney fragment was used as a negative control. Kidney epithelial cells from animals infected with UPEC-46 and UPEC-46::*afp*A (pPAS3) showed some cellular disorganization and partial detachment of the kidney epithelia, but no bacterial adherence was observed. No tissue alterations were present in organs infected by mutant strains.
